# Bayesian variable selection logistic regression with paired proteomic measurements

**DOI:** 10.1002/bimj.201700182

**Published:** 2018-06-25

**Authors:** Alexia Kakourou, Bart Mertens

**Affiliations:** ^1^ Department of Medical Statistics and Bioinformatics Leiden University Medical Center 2300 RC Leiden The Netherlands

**Keywords:** added‐value assessment, Bayesian variable selection, isotope clusters, mass spectrometry, paired measurements, prediction

## Abstract

We explore the problem of variable selection in a case‐control setting with mass spectrometry proteomic data consisting of paired measurements. Each pair corresponds to a distinct isotope cluster and each component within pair represents a summary of isotopic expression based on either the intensity or the shape of the cluster. Our objective is to identify a collection of isotope clusters associated with the disease outcome and at the same time assess the predictive added‐value of shape beyond intensity while maintaining predictive performance. We propose a Bayesian model that exploits the paired structure of our data and utilizes prior information on the relative predictive power of each source by introducing multiple layers of selection. This allows us to make simultaneous inference on which are the most informative pairs and for which—and to what extent—shape has a complementary value in separating the two groups. We evaluate the Bayesian model on pancreatic cancer data. Results from the fitted model show that most predictive potential is achieved with a subset of just six (out of 1289) pairs while the contribution of the intensity components is much higher than the shape components. To demonstrate how the method behaves under a controlled setting we consider a simulation study. Results from this study indicate that the proposed approach can successfully select the truly predictive pairs and accurately estimate the effects of both components although, in some cases, the model tends to overestimate the inclusion probability of the second component.

## 1 INTRODUCTION

Proteomics is the large‐scale study of proteins that aim to provide a better understanding of the function of cellular and disease processes at the protein level. The most widely used technology to assess proteomic expression is mass spectrometry that has undergone remarkable evolution over the last 20 years. Particularly ultrahigh‐resolution mass spectrometers (MS) such as Fourier‐transform MS have become the most powerful and efficient tools for the quantitative analysis of complex protein mixtures in biological systems.

**Figure 1:**
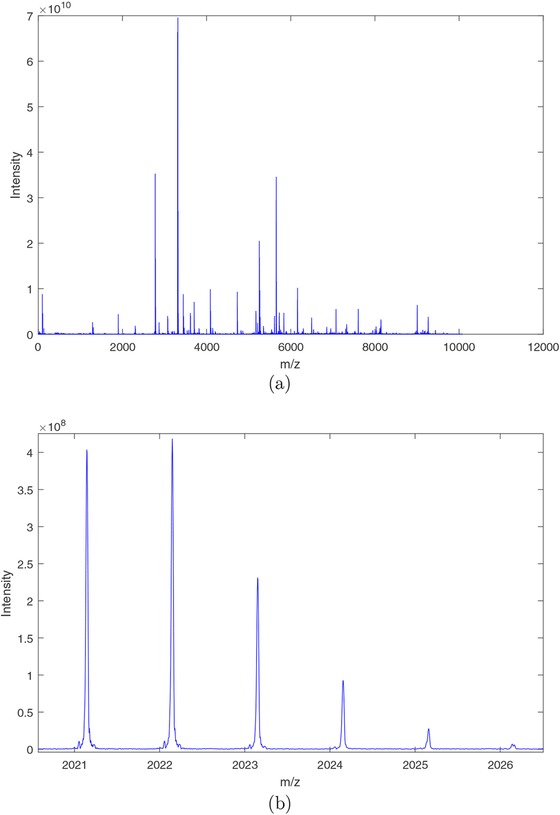
(A) A mass spectrum for a single individual. (B) An isotopic cluster at position *m/z* 2021,2

Irrespective of its type, a mass‐spectrometer takes as input a molecular mixture and outputs a so‐called mass spectrum. A mass spectrum (as shown in Figure 1A) is a sequence of intensity readings distributed over a mass‐to‐charge (*m/z*) range, generated from the detection of ionized molecules. In ultrahigh‐resolution mass spectrometry, each species (such as peptide) is detected and expressed as a “density” of isotope peaks (as shown in Figure 1B)—rather than a single peak—in the mass spectrum, resulting from the distribution of naturally occurring elements. The peaks of an isotopic density represent ions of the same elemental composition but different isotopic composition due to the presence of additional neutrons in their nucleus. We commonly refer to these isotopic densities as isotopic clusters.

The late improvements in mass spectrometry technologies, and thus the quality of the acquired data, turned the focus of recent research toward methods for optimally processing and interpreting high‐resolution mass spectral data (at the individual level) using knowledge on the properties of the isotope densities (such as peak detection algorithms or deisotoping methods). However, the predictive potential of the isotopic cluster information, inherent in such data, has not been fully exploited yet. At date, a common practice for summarizing the proteomic expression (for example so that it can be later used for prediction purposes) is to use information on the amount of expression (intensity).

In a recent work, Kakourou, Vach, Nicolardi, van der Burgt, and Mertens (2016) proposed an alternative approach to using the relative intensity level for summarizing the predictive information within isotope clusters in individual mass spectra. Recognizing that an isotope cluster is a density, they proposed to use, apart from information on the intensity, information on the shape of the isotopic clusters in order to translate the isotopic expression into cluster summaries that could be later used as new predictors for the construction of diagnostic rules. Shape‐based summary measures aim to estimate the shape of either directly the observed isotopic cluster pattern or, alternatively, the deviation of that observed pattern from the typical pattern (as defined in Kakourou et al., 2016). Using the proposed summary measures (based on either intensity or shape information) as new input variables (separately) into a prediction model, the authors showed that both types of information are predictive of the health status of an individual, though intensity has greater predictive capacity as compared to shape.

Having established the presence of an overall isotope cluster effect as well as a shape effect in addition to intensity effect, our aim in this work is twofold: (a) to identify a collection of isotope clusters associated with the disease outcome and (b) to optimally integrate the two sources of information. We wish to address these questions in a way that allows us to assess the added‐value of shape beyond intensity in predicting the class outcome of an individual while maintaining predictive performance.

Several approaches have been proposed in the literature to address the problem of variable selection in the prediction of binary outcomes, with Lasso regularization (Tibshirani, 1996) being among the most popular ones. The lasso uses a *l*_1_‐norm constraint on the vector of regression coefficients that shrinks the parameter estimates toward zero while it induces sparsity in the model. An extension of the lasso, introduced as “group lasso” was proposed (Meier, van de Geer, and Bhlmann, 2008) to perform variable selection on (predefined) groups of variables in logistic regression models. This procedure is a regularization algorithm that acts like the lasso at the group level. Depending on the amount of regularization, an entire group of predictors may drop out of the model. If the groups are all of size one, the group lasso reduces to the lasso. A key limitation of the group lasso is that it does not yield sparsity within a group. That is, if a group of variables is included in the model then all variables within that group are attributed a nonzero effect. For problems with grouped covariates, Simon, Friedman, Hastie, and Tibshirani (2012) considered a more general penalty, termed “sparse‐group lasso,” which yields sparsity at both the group and individual variables levels, in order to select groups as well as predictors within a group. The proposed penalty is a convex combination of the lasso and group lasso penalties that results in both “group‐wise sparsity” and “within‐group sparsity.”

While the sparse‐group lasso has been reported to give favorable results in prediction and classification problems, it selects within‐group predictors without taking into account any prior knowledge on the relative importance of each individual predictor in the group. Given that such prior (or expert) knowledge exists, a more suitable/interesting approach would be to introduce some type of hierarchy in the selection process, for example by prioritizing selection of specific predictors that have already proven their predictive value in previous applications. This would additionally allow us to assess the complementary predictive potential of “secondary” predictors after accounting for “primary” predictors (the term is used with respect to their established predictive capacity, cost of measuring etc.).

The problem of assessing the added‐value of a secondary predictor on top of a primary predictor in the high‐dimensional setting has been considered by Boulesteix and Hothorn (2010) and by Rodríguez‐Girondo et al. (in press). Boulesteix and Hothorn proposed a permutation‐based testing procedure for assessing the additional predictive value of high‐dimensional molecular data on top of clinical predictors through combinations of logistic regression and boosting methods. Rodríguez‐Girondo et al. introduced a two‐stage approach for the assessment of the added predictive ability of omic predictors, based on sequential cross‐validation and regularized regression models. However, both of these approaches are used to evaluate whether there is additional predictive information in a secondary source after correcting for a primary source at a “global level,” hence they do not address the question of where does the extra predictive information come from (which specific predictors within the secondary source, if any, carry additional information on the outcome) or how large is the additional contribution of any individual predictor from the secondary source.

In this paper, we propose an approach that can explore the problem of isotope selection and at the same time address the problem of assessing the additional predictive value of the shape source over and above the intensity source. Our approach uses a Bayesian model formulation that exploits the paired structure of the proteomic data and utilizes prior knowledge on the relative predictive power of each individual source to assess the added‐value of shape, by introducing multiple layers of selection. In doing so, the Bayesian model makes the explicit assumption that the shape source is complementary. In terms of model fitting, this is translated by assuming that a shape measure can be included in the set of predictors on the condition that it is accompanied by/coupled with its corresponding intensity while the reverse does not need to hold. This assumption allows us to make simultaneous inference on which isotope clusters are the most informative with respect to the class outcome and for which isotopes—and most importantly to what extent—shape has a complementary value in separating the two groups.

The remainder of this paper is organized as follows: We first introduce the pancreatic cancer data and their paired structure that is a key component of these particular data. The problem of isotope selection, on the one hand, and assessment of the added‐value of shape, on the other, through a Bayesian model formulation is explored next. We then present results from applying the Bayesian model to the pancreatic cancer data consisting of the paired intensity‐shape measurements. Results from the analysis of the fitted model show that most predictive potential is achieved with a subset of just 6 (out of 1289) pairs, which are consistently selected with very high probability of inclusion, while the contribution of the intensity components is much higher than the shape components. We explore the relative performance and the degree of agreement (with respect to the selected isotope clusters) between our method and an alternative approach based on a stability selection strategy utilizing sparse‐group lasso. Additionally, we show a simulation example to demonstrate how the proposed method behaves under a controlled setting. The outcome of the simulation study indicates that the proposed approach can successfully select the truly predictive pairs and accurately estimate the effects of both components although, in some cases, the model tends to overestimate the inclusion probability of the second component. We finish with a discussion.

## 2 DATA

In this paper, we reanalyze data from a case‐control study that was carried out at the Leiden University Medical Centre (Nicolardi et al., 2014). The study involved 273 individuals, consisting of 88 pancreatic cancer patients and 185 healthy volunteers. The samples collected from those individuals were distributed over three MALDI‐target plates and thereafter mass analyzed by a MALDI‐FTICR MS system, giving rise to a single mass spectrum for each sample within the mass range of 1013–3700 Da (full details on the design and measurement protocol can be found in Nicolardi et al., 2014).

In previous work (Kakourou et al., 2016), the authors applied to the pancreatic cancer data a peak detection algorithm in order to identify the isotopic clusters and their corresponding peaks. As a result, the complete proteomic expression in the individual spectra was reduced to clusters of isotopic expression on which summary measures could be defined. To derive the cluster summaries the authors proposed to use information on either the intensity or the shape of the observed isotope cluster pattern.

In this work, rather than considering single measurements of intensity or shape as our predictor variables, we recognize intensity and shape are tied together and regard them as paired measurements such that the components of each predictor pair represent cluster summaries of isotopic expression based on intensity information, in the case of the first component, and shape information, in the case of the second. The intensity component of a pair is denoted in the following by *u* and is defined as the sum of log‐transformed peak intensities $l_{j},j=1,\ldots ,J$ within an isotope cluster (where *J* denotes the number of peaks in a cluster and is cluster‐specific), given by

(1) $$u:=\sum_{\begin{array}{c} j \end{array}}l_{j}$$

The shape component, denoted by *v*, is defined as

(2) $$v:=\sum_{\begin{array}{c} j \end{array}}jp_{j}$$

the center of gravity of a distribution on the values 1,…,J, where $p_{j}:=\frac{x_{j}}{\sum_{\begin{array}{c} j \end{array}}x_{j}}$ and $x_{1},\ldots ,x_{J}$ denote the residuals that measure the deviation of the observed isotopic pattern from the typical pattern. For a more detailed description on how these summary measures were created/derived we refer the interested readers to Kakourou et al. (2016). We choose these particular summary measures to represent the intensity‐based and shape‐based components due to their superiority, as compared to other proposed summary measures, with regard to their individual predictive ability.

Our final dataset consists of 1289 pairs of intensity and shape summary measures. Our objective is to investigate whether we can develop methods to integrate both types of information in a way that will allow us to calibrate more interpretable rules and/or learn more about the interplay between intensity and shape in predicting the class outcome, while maintaining predictive performance.

## 3 BAYESIAN VARIABLE‐SELECTION MODEL ON PAIRS

### 3.1 The logistic regression model

Let the data be given by (y,z), where $y={\left(y_{1},\ldots ,y_{n}\right)}^{⊺}$ is the binary case‐control outcome with $y_{i}\in \left\{0,1\right\}$ for i=1,…,n independent individuals while $z={\left(z_{1},\ldots ,z_{n}\right)}^{⊺}$ represents the predictor source with each $z_{i}=\left(\left(u_{i1},v_{i1}\right),\ldots ,\left(u_{ip},v_{ip}\right)\right)$ consisting of a sequence of paired intensity and shape measurements for *p* isotope clusters. We consider the binary regression model

(3) $$y_{i}∼\text{Benoulli}\left(p_{i}\right)$$

with

(4) $$\text{logit}\left(p_{i}\right)=\beta_{0}+z_{i}\beta$$

where $p_{i}$ is the case‐probability for the *i*‐th observation and $\beta =(\left(a_{1},b_{1}\right),\ldots ,\left(a_{p},b_{p}\right))$ represents the vector of paired regression parameters with the first and second elements of each pair corresponding to the effects of the intensity and shape measurements respectively.

### 3.2 Variable‐dimension logistic regression model

We assume that only a subset of isotope clusters is relevant for predicting the class outcome and that u is expected to carry more information on the class outcome than v. Our main objective is to assess the added‐value of the shape source v on top of the intensity source u in predicting the health status of future patients. Under the assumption that only a set of isotope clusters is predictive of the health status of an individual, the true model is given by

(5) $$\begin{array}{cc} \text{logit}(p_{i}) & =\beta_{0}+{\tilde{z}}_{i}\tilde{\beta }=\\ & =\beta_{0}+\left({\tilde{a}}_{1}{\tilde{u}}_{i1}+{\tilde{b}}_{1}{\tilde{v}}_{i1}\right)+\left({\tilde{a}}_{2}{\tilde{u}}_{i2}+{\tilde{b}}_{2}{\tilde{v}}_{i2}\right)+\cdots +\left({\tilde{a}}_{k}{\tilde{u}}_{ik}+{\tilde{b}}_{k}{\tilde{v}}_{ik}\right)=\\ & =\beta_{0}+\sum_{j=1}^{k}\left({\tilde{a}}_{j}{\tilde{u}}_{ij}+{\tilde{b}}_{j}{\tilde{v}}_{ij}\right) \end{array}$$

where $\tilde{z}=\left(\left({\tilde{u}}_{1},{\tilde{v}}_{1}\right),\ldots ,\left({\tilde{u}}_{k},{\tilde{v}}_{k}\right)\right)$ represents the unknown set of paired measurements that are associated with the class outcome with regression coefficient vector $\tilde{\beta }=\left(\left({\tilde{a}}_{1},{\tilde{b}}_{1}\right),\ldots ,\left({\tilde{a}}_{k},{\tilde{b}}_{k}\right)\right)$ that is of unknown dimension k≤p. The isotope dimensionality *k* is thus considered/treated as a parameter in the model and will be estimated from the data along with the unknown set of intensity‐shape pairs and their corresponding regression coefficients.

In order to assess the added‐value of shape information over and above intensity information we place a logical constraint on the inclusion of shape information that specifies that if a=0 then b=0 (if b≠0 then a≠0). The above formulation suggests that shape can be included in the model only on the condition that its corresponding intensity is included as well. Note that the reverse is not true. Hence, rather than forcing mutual selection of both components of the isotope pairs we let the data decide whether the first component (intensity) alone provides all the required information for separating the two groups. With this constraint, (5) reduces to

(6) $$\text{logit}\left(p_{i}\right)=\beta_{0}+\sum_{j=1}^{k_{C}}\left({\tilde{a}}_{j}{\tilde{u}}_{ij}+{\tilde{b}}_{j}{\tilde{v}}_{ij}\right)+\sum_{l=1}^{k_{I}}{\tilde{a}}_{l}{\tilde{u}}_{il}$$

where $k_{C}$ denotes the dimensionality of the “complete” isotope couples and indicates how many times a shape measure is included in the model in conjunction with its corresponding intensity and $k_{I}$ denotes the dimensionality of intensity singletons so that $k=k_{I}+k_{C}$.

### 3.3 Prior specification

To complete the model formulation, we have to specify the prior structure for all the model parameters. For the intercept we assume a weakly informative normal prior $\beta_{0}∼N\left(0,10^{2}\right)$. We specify independent normal priors on the regression parameters ${\tilde{a}}_{j}∼N\left(0,\sigma_{a}^{2}\right)$ and ${\tilde{b}}_{j}∼N\left(0,\sigma_{b}^{2}\right)$, for j=1,…,k and $b_{j}\neq 0$, where the variances $\sigma_{a}^{2}=\tau_{a}^{2}c_{a}$ and $\sigma_{b}^{2}=\tau_{b}^{2}c_{b}$ control the magnitude of included effects for intensity and shape respectively, $\tau_{a}$ and $\tau_{b}$ are known rescaling factors while $c_{a}=1/s_{a}$ and $c_{b}=1/s_{b}$ are randomly distributed scale factors with gamma priors placed on $s_{a}$ and $s_{b}$. Under the above prior assumption on the regression parameters, the covariance matrix Σ has a block‐diagonal structure with block matrices along the diagonal of the form $\Sigma_{j}=[\begin{array}{cc} \sigma_{a}^{2} & 0\\ 0 & \sigma_{b}^{2} \end{array}]$, if $b_{j}\neq 0$, and $\Sigma_{j}=\sigma_{a}^{2}$ otherwise. The prior specification is completed by assigning a prior to the isotope dimension parameter *k*. We use a discrete uniform prior on the set of integers $\left\{0,1,2,\ldots ,k_{max}\right\}$ with $k_{max}$ a large positive integer corresponding to the maximum allowed isotope dimension.

### 3.4 MCMC model fitting

We present an adaptation of the reversible jump MCMC implementation described in Mertens (2016) for fitting the logistic model, in order to perform isotope selection, on the one hand, and assess the added‐value of shape beyond intensity, on the other. Evaluation of the added‐value of shape is achieved by introducing a second layer of shape selection on top of the isotope selection.

Our MCMC sampler is based on a random choice between three basic move steps: (1) BIRTH, (2) DEATH, and (3) CHANGE. The first two of these steps propose moves between different isotope dimensions while the last one proposes moves within an isotope dimension and between different variable dimensions.

More specifically, in the BIRTH step, we propose with probability $b_{k}=1/3$ to add a new randomly chosen isotope into the model set. In the DEATH step, we propose with probability $d_{k}=1/3$ to remove a randomly chosen isotope from the current model set. Shape selection is facilitated by splitting each of these steps into two additional substeps such that, in the case of a BIRTH move proposal, we may choose between proposing to add either the entire pair (couple) into the model, with probability $b_{k}^{C}=b_{k}/2$, or solely the first component of the pair (intensity), with probability $b_{k}^{I}=b_{k}/2$. Note that the candidate set from which we may select a new isotope is the set containing all “complete” isotopes that do not currently have any component in the model set. Analogously, in the case of a DEATH move proposal, we may choose between proposing to remove either a complete isotope (couple) from the current set, with probability $d_{k}^{C}=d_{k}/2$, or an intensity singleton, with probability $d_{k}^{I}=d_{k}/2$.

Apart from the BIRTH and DEATH steps that propose moves between isotope dimensions by either adding or removing isotopes (singletons or pairs) to and from the current set, we may propose to change the composition of an isotope already included in the model, with probability $c_{k}=1/3$. We do so by introducing a CHANGE step. Again here, within this step, we may choose between two substeps that either change an isotope couple into an intensity singleton that is remove shape from the included isotope pair, with probability $c_{k}^{C\to I}=c_{k}/2$, or change an intensity singleton into an isotope couple that is add shape to the included intensity singleton, with probability $c_{k}^{I\to C}=c_{k}/2$. In this way, we give a second chance to shape selection/deselection by allowing the data to judge whether a shape measure contributes to classification in addition to intensity and thus should join its corresponding intensity or does not provide any additional information over and above intensity and therefore could be omitted from the isotope pair in the model set.

We choose

$$\begin{array}{ccc} & & b_{\left(k=0\right)}^{I}=b_{\left(k=0\right)}^{C}=d_{\left(k=k_{max}=k_{I}\right)}^{I}=d_{\left(k=k_{max}=k_{C}\right)}^{C}=c_{\left(k=k_{max}=k_{I}\right)}^{I\to C}=c_{\left(k=k_{max}=k_{C}\right)}^{C\to I}=1/2,\\ & & d_{\left(0<k_{I},k_{C}<k_{max}=k\right)}^{I}=d_{\left(0<k_{I},k_{C}<k_{max}=k\right)}^{C}=c_{\left(0<k_{I},k_{C}<k_{max}=k\right)}^{C\to I}=c_{\left(0<k_{I},k_{C}<k_{max}=k\right)}^{I\to C}=1/4,\\ & & b_{\left(k_{I}=0<k_{C}<k_{max}\right)}^{I}=b_{\left(k_{I}=0<k_{C}<k_{max}\right)}^{C}=d_{\left(k_{I}=0<k_{C}<k_{max}\right)}^{C}=c_{\left(k_{I}=0<k_{C}<k_{max}\right)}^{C\to I}=1/4,\\ & & b_{\left(k_{C}=0<k_{I}<k_{max}\right)}^{I}=b_{\left(k_{C}=0<k_{I}<k_{max}\right)}^{C}=d_{\left(k_{C}=0<k_{I}<k_{max}\right)}^{I}=c_{\left(k_{C}=0<k_{I}<k_{max}\right)}^{I\to C}=1/4,\\ & & b_{\left(k=k_{max}\right)}^{I}=b_{\left(k=k_{max}\right)}^{C}=d_{\left(k_{I}=0\right)}^{I}=d_{\left(k_{C}=0\right)}^{C}=c_{\left(k_{C}=0\right)}^{C\to I}=c_{\left(k_{I}=0\right)}^{I\to C}=0 \end{array}$$

and

$$b_{k}^{I}=b_{k}^{C}=d_{k}^{I}=d_{k}^{C}=c_{k}^{C\to I}=c_{k}^{I\to C}=1/6$$

in all other cases. Under this specification, the acceptance probability of a Metropolis–Hastings step for a proposal move from a model with parameters θ to a new model with parameters $\theta^{\prime }$ is

(7) $$A=\min\left\{1,\frac{P(D|\theta^{\prime })}{P(D|\theta )}\frac{p(\theta^{\prime })}{p(\theta )}\frac{q(\theta |\theta^{\prime })}{q(\theta^{\prime }|\theta )}\right\},$$

the ratio of marginal likelihoods of the newly proposed model to that of the old, prior, and proposal distributions, with *D* the data and θ the vector containing the current values of the model parameters *k*, $\sigma_{a}^{2}$ , and $\sigma_{b}^{2}$, (see Appendix for detailed derivation of acceptance probabilities for all move types).

Note that with the above prior specification, normal inverse scaled chi‐squared updating can be applied, conditional on the variance hyper‐parameters. This implies that the “conditional” acceptance rates do not require actual calculation of the regression parameters on either the old or newly proposed models, as closed forms are available for the integrated likelihood functions conditional on the above variance terms. This leads to significant gains in computational speed. We use the auxiliary variable construction with Kolmogorov–Smirnow prior on the normal variance to obtain the logistic regression model form.

We present the basic structure of the algorithm in form of pseudo code below:

Algorithm 1 **RJ sampler**

1

**Table:** 

Set $\theta^{\left(0\right)}=\left(\right);$
Set t=0;
REPEAT
Draw *u*_1_ from a *U*(0, 1) distribution
**If** $u_{1}\leq b_{k}$ propose a ***birth step***
**if** $u_{1}\leq b_{k}/2$
$\theta^{\prime }=\text{birth}\;\text{of}\;\text{intensity}$ ***singleton*** −proposal;
**else**
$\theta^{\prime }=\text{birth}\;\text{of}\;\text{intensity-shape}$ ***pair*** −proposal;
**end;**
**else if** $b_{k}\leq u_{1}\leq b_{k}+d_{k}$ propose a ***death step***
**if** $u_{1}\leq b_{k}+d_{k}/2$
$\theta^{\prime }=\text{death}\;\text{of}\;\text{intensity}$ ***singleton*** −proposal;
**else**
$\theta^{\prime }=\text{death}\;\text{of}\;\text{intensity-shape}$ ***pair*** −proposal;
**end;**
**else** propose a ***change step***
**if** $u_{1}\leq b_{k}+d_{k}+c_{k}/2$
$\theta^{\prime }=\text{change}\;\text{of}$ ***singleton*** *to* ***pair*** −proposal;
**else**
$\theta^{\prime }=\text{change}\;\text{of}$ ***pair*** *to* ***singleton*** −proposal;
**end;**
**End;**
Draw *u*_2_ from a *U*(0, 1) distribution
**If** $u_{2}<\min\left\{1,A\right\}$
$\theta^{\left(t+1\right)}=\theta^{\prime }$;
**else**
$\theta^{\left(t+1\right)}=\theta^{\left(t\right)}$;
**End;**
t=t+1;
Store every *m*th value of $\theta^{\left(t\right)}$ after initial burn‐in;
END REPEAT;

## 4 RESULTS

### 4.1 Application

We applied the isotope variable‐dimension model to the pancreatic cancer data that contained 2578 intensity and shape measurements in total, coupled in 1289 isotope pairs. For the maximum allowed isotope dimensionality we used $k_{max}=100$. We set the hyperparamaters of the Gamma distribution to α=β=1 for the analysis presented in the paper, which results in a prior mean and variance of 1 for both $s_{a}$ and $s_{b}$.

For the prior choice on the scaling factors $\tau_{a}$ and $\tau_{b}$ we make use of the knowledge that the variance‐covariance matrix of the regression parameter vector in the logistic regression model is approximately $n*{\left(X^{T}X\right)}^{-1}$, where *n* is the sample size and X is the data matrix. We find that the inverse cross‐products of $u^{T}u$, where $u=(u_{1},\ldots ,u_{k})$ and $v^{T}v$, where $v=(v_{1},\ldots ,v_{k})$, are of order 10^−3^ and 10^−2^ which, for a sample size of n=254, points to a prior guess of 0.25 and 2.5 for $\tau_{a}$ and $\tau_{b}$, respectively.

### 4.2 Convergence

To perform the isotope selection, we sampled two sets of 500,000 simulations, after an initial burn‐in of 200,000 samples that were discarded. Convergence was assessed by comparing the last set of simulations with the first through autocorrelation and kernel density plots on the model parameters such as the isotope dimensionality *k*, the intensity‐shape pair and intensity singleton dimensionalities $k_{C}$ and $k_{I}$, the regression parameters as well as the model variances for intensity and shape over the MCMC chains. For posterior inference on the model we combine the two sets of updates into a single set of simulations (1 million samples).

### 4.3 Post‐hoc analysis

**Figure 2:**
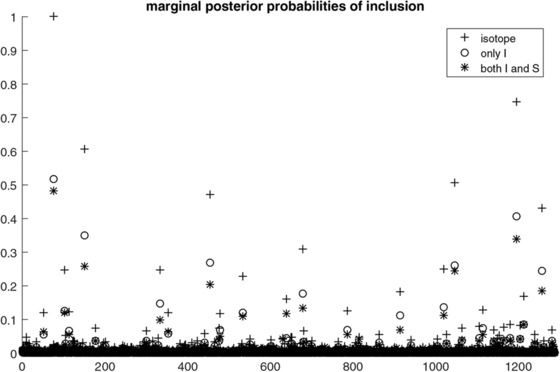
Posterior probabilities of inclusion into the model for any specific isotope configuration (crosses), intensity component alone (circles) and both intensity and shape components (asterisks) versus the isotope cluster number

Previous results on the pancreatic cancer data showed evidence for the presence of predictive information in the isotope clusters that can be used for diagnostic purposes. More specifically, results from using isotope cluster summaries based on either intensity or shape information as input into a ridge logistic model independently, showed that both types of information are predictive of the class outcome, though intensity was found to be more informative than shape. Having established the presence of predictive capacity in the proteomic data, our focus turns toward identifying a collection of isotope clusters associated with the disease as well as investigating the predictive value of shape beyond intensity within the isotope clusters. We begin our exploration by investigating how often an isotope was selected in the model as well as the selection frequency of isotopes consisting of intensity alone and of isotopes consisting of both intensity and shape measures. Figure 2 shows the marginal probabilities of inclusion into the model for each of the 1289 isotopes plotted against the isotope cluster number. Crosses correspond to marginal posterior probabilities of inclusion of any specific isotope configuration, irrespective of its composition, circles correspond to marginal posterior probabilities of inclusion of the intensity component alone while asterisks correspond to marginal posterior probabilities of inclusion of both the intensity and shape components such that the value of any cross equals the sum of the values of the circles and asterisks. As can be seen, there is rather strong evidence in favor of specific isotopes. In particular, isotope 77 is selected into the model with a probability of almost one while half of the times the model selects both intensity and shape measures to be included in the set of predictors. We observe a general tendency of the model to select both intensity and shape measures almost as often as intensity alone. This is particularly true for the isotopes with high probability of inclusion.

**Figure 3:**
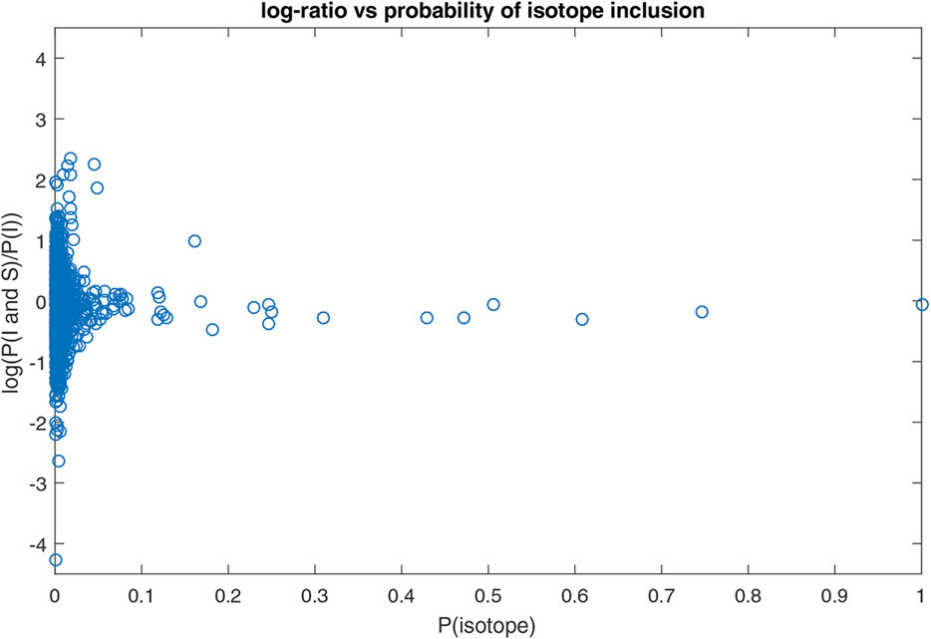
Estimates of ratio of inclusion probabilities for both intensity & shape and intensity only (on the log‐scale) versus probability of isotope inclusion

This tendency of the model is more apparent in Figure 3 where we plot the ratio estimates of inclusion probabilities on the log scale, that is the ratio between the probability of the entire intensity‐shape pair to be selected and the probability of intensity alone to be selected in the model, versus the probabilities of isotope inclusion. We notice that the isotopes for which mutual intensity and shape selection is more frequent than individual intensity selection (log‐ratio estimates of inclusion probability above 0) are the ones with the lowest overall (isotope) probability of inclusion. On the other hand, the log‐ratio estimates of the isotopes with the highest probabilities of inclusion are close to zero (ratio close to one) that means that for those isotopes the model selects both components with the same frequency that it selects the first component alone. This outcome suggests that in those cases, the model cannot distinguish between including or excluding shape to or from the model. In general, we observe that the log‐ratio estimates get closer to zero for increasing values of the overall isotope inclusion probability.

**Figure 4:**
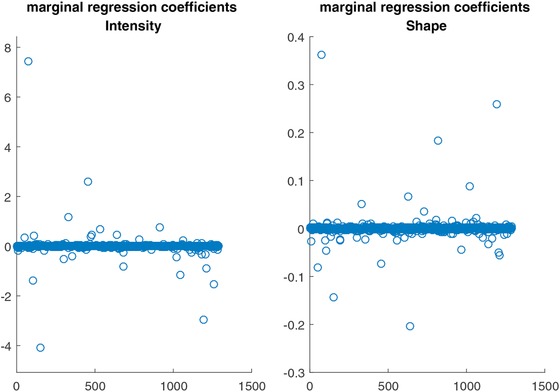
Marginal mean regression coefficients of the Bayesian model for intensity (left plot) and shape (right plot) versus the isotope cluster number

The selection effect is reflected, apart from the selection itself, in the calibration of the regression coefficients. We further investigate the effect of selecting intensity or shape and their relative contribution to classification by calculating the marginal mean of regression coefficients as

(8) $$\sum_{m\in M}\beta_{m}/M,$$

with *m* denoting the current model within the set of all simulated models and *M* denoting the total number of simulated models. Figure 4 plots these marginal mean regression coefficients separately for intensity (left plot) and shape (right plot) across all simulated models. We can see from these plots that shape is associated with effects of much lower magnitude, as compared to intensity, despite the relatively high frequency with which certain shape measures were selected in the model. This outcome may confirm our intuition that the intensity source carries more information on the class outcome than the shape source, however we must be careful when comparing the shape effects with the intensity effects since the observed differences in effect magnitude could be due to the systematic scale differences between the intensity and shape summary measures.

Essentially, the selection effect is shared between two types of parameters: (1) the inclusion probability and (2) the regression effect. To get additional insights into the relative contribution of intensity and shape we define a “combined” Posterior Summary (PS), which integrates the information from the effect of inclusion and the calibrated regression effect, as

(9) $$PS=\sum_{m\in M}\left(\right|\beta_{m}|/\sum_{j=1}^{k^{{}^{\prime }}}\left|\beta_{m_{j}}\right|)(1/k^{{}^{\prime }})/M,$$

where $\sum_{j=1}^{k^{{}^{\prime }}}\left|\beta_{m_{j}}\right|$ is only across either all intensity or all shapes measures, with $k^{{}^{\prime }}$ the total number of intensity measurements (equal to $k_{I}+k_{C}$) or the total number of shape measurements (equal to $k_{C}$) selected in model m∈M, depending on whether PS is calculated for an intensity or a shape measure, respectively. We choose to compute PS separately for intensity and shape measures as a way to account for the different scales of the two predictor sources.

**Figure 5:**
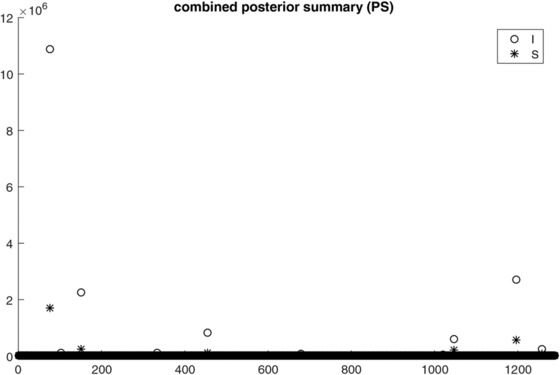
Combined posterior summary estimates for intensity (circles) and shape (asterisks) versus the isotope cluster number

**Figure 6:**
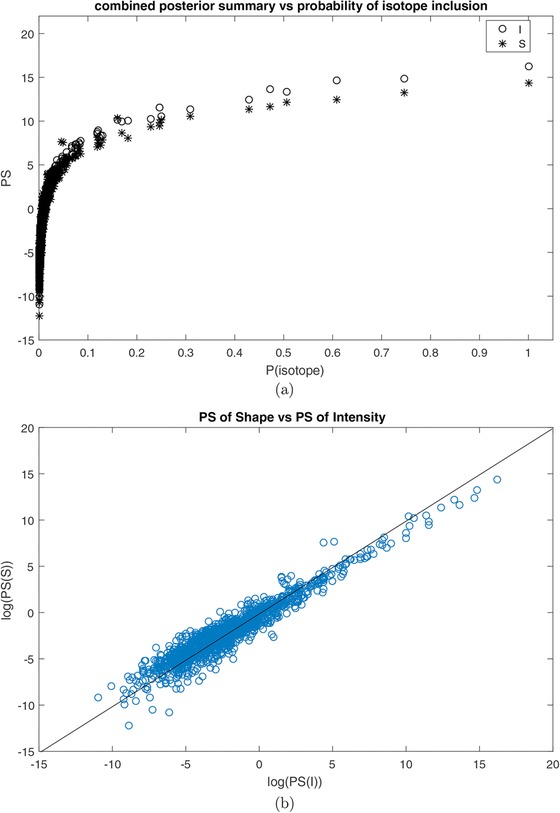
(A) Combined posterior summary (on the log scale) of intensity (circles) and shape (asterisks) versus probability of isotope inclusion. (B) Combined posterior summary (on the log scale) of intensity versus combined posterior summary (on the log scale) of shape

The combined posterior summary measures for intensity (circles) and shape (asterisks) are shown in Figure 5 from which it can be seen that there are in total six pairs that stand out as the most important predictor couples while the intensity components of these pairs have higher importance/contribution than their corresponding shape components. In the top plot of Figure 6, we plot the log‐transformed combined posterior summaries for intensity (circles) and shape (asterisks) against the probability of isotope inclusion to get a more clear picture of the relative contribution of the two measures. Again here, we see six circles and six asterisks standing out that correspond to the most important intensity and shape measures according to the combined posterior summary while we see that effectively most asterisks representing the shape components lie continually below the circles representing the intensity components, especially for the pairs with large values of probability of isotope inclusion. Similar conclusions can be drawn when looking at the bottom plot of Figure 6, which shows the log‐transformed combined posterior summaries of intensity versus the log‐transformed combined posterior summaries of shape. For the pairs with the most important intensity components (according to their PS estimates), the PS values of the shape components are consistently smaller than those of the intensity components.

### 4.4 Stability selection with sparse‐group lasso

We compare results based on our Bayesian variable selection approach against results obtained with an alternative frequentist variable selection approach based on a stability selection strategy that utilizes sparse‐group lasso (SS‐SGL). Stability selection aims to provide a measure of confidence in the selection of variables reported by sparse‐group lasso by adding a resampling step that involves repeating the variable selection procedure over a number of datasets, randomly sampled from the original. We partition the original data into 10, approximately equal sized, subsamples. For each subsample analysis, we discard one subsample in turn and record the components selected by the sparse‐group lasso fitted on the remaining nine subsamples. We define the “selection probabilities” calculated across the results of all subsampled datasets. Intuitively, the selection probabilities can be considered equivalent to the inclusion probabilities generated by our Bayesian variable selection approach and provide a measure of certainty for each selected component since selection of the most informative predictors should be more robust to random perturbations of the data.

It should be noted that the selection probabilities resulting from SS‐SGL do not have the same interpretation as posterior probabilities. Moreover, the definition of these probabilities is different from that of the inclusion probabilities because of the conditional nature of the Bayesian selection approach, since it selects shape components on the condition that their corresponding intensity components are included in the model. This asymentrical way of selecting components is introduced due to the underlying assumptions/hypotheses linked with prior knowledge about the relative contribution of shape with respect to intensity. As a result, the model spaces explored by our approach and SS‐SGL are different, leading to interpretational differences. Therefore, a comparative analysis can be carried out solely with respect to the degree of concordance and discordance between the two methods regarding the sets of selected isotopes as well as their relative predictive performance.

Table 1 shows percentiles of the selected isotope dimensionalities with our Bayesian approach (first row) and SS‐SGL (second row). We observe that sparse‐group lasso tends to select higher dimensional models to describe the data as compared to our Bayesian selection approach. In particular, the minimum number of isotope clusters selected by our Bayesian approach is four while sparse‐group lasso selects a minimum of 59 clusters, 19 of which are due to selection of shape alone. The number of clusters selected by SS‐SGL due to shape are shown in the last row of Table 1. Note that such isotope cluster configurations are not allowed to enter the model with our Bayesian approach.

**Table 1:** Percentiles of isotope dimensionality based on the Bayesian variable selection model (first row), stability selection with sparse‐group lasso (SS‐SGL) (second row) and stability selection with sparse‐group lasso due to shape (last row)

Percentile	min	1.0	2.5	5.0	25	50	75	90
nr of selected isotopes (Bayesian model)	4	5	6	7	12	15	19	23
nr of selected isotopes (SS‐SGL)	59	59	59	59	66	74	83	94
nr of selected isotopes due to shape (SS‐SGL)	19	19	19	19	21	30	33	38

**Figure 7:**
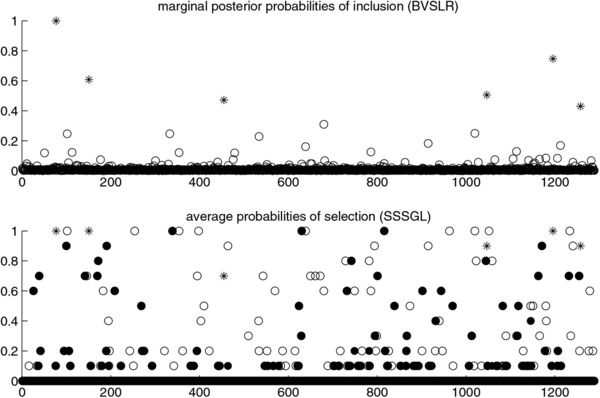
Posterior probabilities of inclusion based on the Bayesian model (upper plot) and selection probabilities based on stability selection with sparse‐group lasso (lower plot) versus the isotope cluster number. Asterisks correspond to the top six isotope clusters selected by the Bayesian approach also selected by sparse‐group lasso. Filled points represent isotope clusters selected by sparse‐group lasso due to shape alone

Figure 7 shows posterior probabilities of inclusion based on the Bayesian variable selection model (upper plot) and selection probabilities based on SS‐SGL (lower plot) versus the isotope cluster number. Asterisks correspond to the top six isotope clusters selected by the Bayesian approach that are also consistently selected by SS‐SGL as indicated by the high values of their corresponding selection probabilities. Filled points represent isotope clusters selected by SS‐SGL due to the shape component alone. It can be seen from this figure that the Bayesian approach favors lower dimensional models and leads to a more consistent variable selection compared to SS‐SGL as suggested by the fairly smaller number of points that lie above the zero probability of inclusion line as well as above the 0.5 probability threshold. It can also be noticed that a large number of isotopes selected by SS‐SGL is due to selection of shape alone (filled points). In particular, there are 27 isotopes selected by SS‐SGL with a probability of at least 0.5 due to shape alone. Note that these isotope configurations can never be included in the model with the Bayesian approach.

### 4.5 Assessment of predictive performance

**Figure 8:**
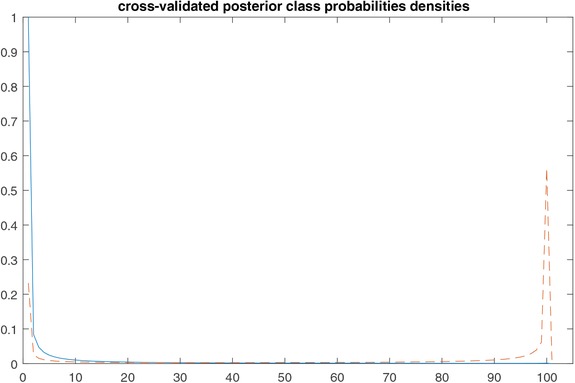
Cross‐validated posterior class probability densities for controls (solid line) and cases (dashed line)

To assess the predictive performance of the isotope dimension model we use 10‐fold internal cross‐validation. We do so by first partitioning the data into 10 mutually exclusive and exhaustive, equal sized, subsamples. We then run 10 parallel chains in each of which one subsample is used as validation data for evaluating the predictive performance of the model, while the remaining nine subsamples are used to calibrate the Bayesian model. Since the case‐control ratio can greatly affect the selection, to get as consistent variable selection and estimates as possible across the different sets, we partition the original data such that the case‐control ratio in the newly defined calibration and validation data is the same across all subsamples. Evaluation of the model on each subsampled validation set is achieved by applying, within each MCMC step in each chain, the generated rule based on the calibration data to the profiles in the validation set and storing the sequence of validated predictions. To assign observations, we calculate for each observation the mean a‐posteriori class probabilities of group‐membership. That is, we compute the cross‐validated probabilities

(10) $$P\left(y_{i}=1|\tilde{u},\tilde{v}\right)=\sum_{m\in M}P_{m}\left(y_{i}=1|\tilde{u},\tilde{v}\right)/M$$

for all i=1,…,n, where $P_{m}$ denotes the a‐posteriori class probability calculated from the *m*‐th model simulated within the MCMC chain and the sum is across all models simulated. To estimate the error rate we use a cut‐off value of 0.5. We assign an observation as a disease case if the mean a‐posteriori class probability is greater than 0.5 and as a control otherwise. This assignment resulted in a misclassification error rate of 0.086. We also calculate the Brier score, defined as

(11) $$B=\frac{1}{n}\sum_{i=1}^{n}{\left(P\left(y_{i}=1|\tilde{u},\tilde{v}\right)-c_{i}\right)}^{2}$$

which equals 0.082, where $c_{i}$ denotes the true class outcome of the *i*‐th individual. Finally, the Area Under the Curve (AUC) was found equal to 0.945 (95% CI [0.915, 0.974]). In Figure 8 we plot the posterior probability densities for the control (solid line) and the case (dashed line) groups. It is worth mentioning at this point that these results are in tune with previously reported results on the pancreatic cancer data. In particular, internally validated results reported in Kakourou et al. (2016), obtained using intensity information across all observed isotope clusters into a ridge logistic model, gave an error‐rate of 0.105 and an AUC of 0.935 (95% CI [0.813, 1]). An additional point to consider is that the validated error rate and AUC based on sparse‐group lasso (where the rule is derived for optimal penalty selected based on cross‐validation on the subsampled calibration sets—as defined above—and results are averaged across subsampled validation sets) were 0.214 and 0.923 (95% CI [0.826, 1]), respectively. This outcome provides some confirmation that incorporating prior knowledge in the analysis by making problem‐specific assumptions, when special (grouped or paired) data structures need to be taken into account, can lead to greater accuracy, as indicated by the difference in error rates between the sparse‐group lasso and our Bayesian approach.

Finally, we investigate the consistency of the model selection across the partitioned datasets and the extent of agreement with the model selection based on the entire data. Table 2 shows the top seven isotope clusters selected into the Bayesian model applied to the full data (second column) in decreasing order of posterior probability of isotope inclusion. The table also gives the rank of the top selected isotope clusters according to the average posterior probability of isotope inclusion calculated across the 10 subsampled data sets (third column) together with these average probability estimates (forth column) and their corresponding standard errors (last column). The top isotope cluster selected by the full model corresponds also to the top isotope cluster selected across submodels with an average probability of 0.87. Nearly all top isotope clusters selected by the full model are identical to the ones selected across submodels, also with identical ranking, except for clusters 1047 and 455 that interchange their ranks as well as cluster 639 that drops from the 7th rank to the 12th, with an average (isotope) inclusion probability of just 0.16.

**Table 2:** Ranks (left column) of top seven selected isotope clusters (second column) based on Bayesian model applied to the full data, rank of top selected isotope clusters according to average probability of isotope inclusion across partitions (third column), average probability estimates of isotope inclusion (forth column), and standard errors of average probability estimates (last column)

Rank (full)	Isotope nr	Rank (average)	P(isotope)	SE
1	77	1	0.87	0.10
2	1196	2	0.50	0.20
3	151	3	0.43	0.13
4	1047	5	0.36	0.15
5	455	4	0.39	0.18
6	1258	6	0.35	0.20
7	639	12	0.16	0.25

### 4.6 A simulation example

We use a simplified simulation example to demonstrate how the method behaves in a controlled setting. We generate data to have the same number of patients as in the pancreatic cancer dataset but smaller dimensionality under the independence assumption. We generate 200 variables in total such that half of them represent the first and the other half the second components of 100 paired measurements. For both components of each pair we simulate data from a normal distribution where we specify the independent normal random variables *u* and *v* as *N*(0, 2.5^2^). We simulate the binary outcome data according to the logistic model

(12) $$\begin{array}{cc} \mathrm{logit}\left(p\right) & =\beta_{0}+\beta z=\\ & =\beta_{0}+\mathrm{au}+\mathrm{bv} \end{array}$$

where a and b are 100‐dimensional vectors containing the first and second component effects on the class outcome. To induce associations between the predictor components and the outcome, we draw binary response variables from a Bernoulli distribution with

(13) $$p=\frac{1}{1+e^{-(\beta_{0}+\mathrm{au}+\mathrm{bv})}}$$

We consider three different scenarios in which we vary the number of true, nonzero effects, as well as their magnitude. In all three scenarios we use $k_{max}=50$, α=β=1 for the hyperparameters of the Gamma distribution and $\tau_{a}=\tau_{b}=1$ while we vary the values of the nonzero elements of β from 1 to 3.5.

**Figure 9:**
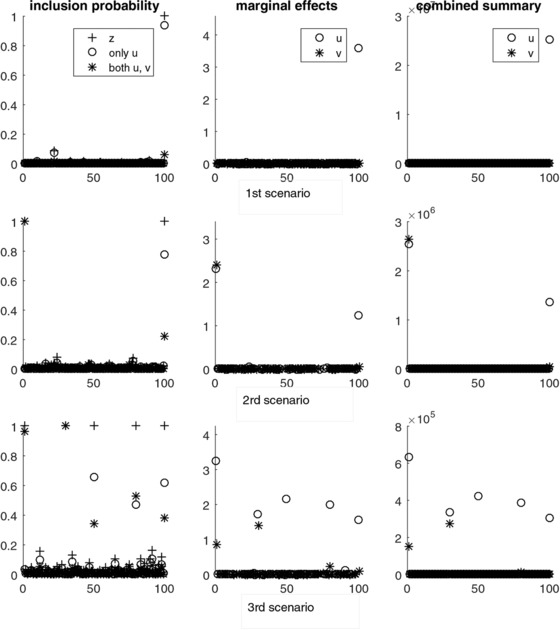
Inclusion probabilities (left plots), marginal regression effects (middle plots), and combined posterior summaries (right plots) of the first (circles) and second (asterisks) components of the pairs for the first (top plots), second (middle plots), and third (bottom plots) scenarios

In the first scenario, we select solely the first component of the last pair to have a nonzero effect and we set its value to be equal to 3.5. We wish to test whether the model can identify the single predictive pair—and more specifically the single predictive component within the pair, on the one hand, and estimate its true effect, on the other. The top three plots of Figure 9 show estimates of the inclusion probabilities, marginal effects, and combined summaries for the first (circles) and second (asterisks) components of the 100 pairs across 200,000 simulations. The key point from these plots is that the model can identify the single discriminating feature corresponding to the first component of the last pair and it can accurately estimate its true effect.

In the second scenario, apart from the first component of the last pair, we assign nonzero effects to both components of the first pair. We set the effect for the two components of the first pair equal to 2.5 and the effect of the first component of the last pair equal to 1.5. Looking at the fist plot in the middle row of Figure 9 that shows the inclusion probability estimates, we observe that the second component of the last pair is selected, alongside with the first, with a probability of about 0.2, although this component does not carry any information on the class outcome. The relative importance of this nonpredictive component is better captured/reflected by the marginal effect estimates and the estimates of combined summary. We see from the two last plots in the middle row of Figure 9 that show these estimates that, although the model selects the noninformative component with a probability 0.2, it assigns it a zero effect, such that the only prominent points in the two plots are the ones that correspond to the three components that are predictive of the outcome. Moreover, we explore the behavior of the Bayesian model under the additional situations in which we have low, moderate or high correlations between the pairs and/or between the predictive components within and across pairs. Results from these investigations were in agreement with results from the no correlations between components case. We include these results in the Appendix.

In the third scenario, we increase the number of pairs with both components discriminative to two and the number of pairs with only the first component discriminative to three such that $a_{k}\neq 0$ for k=1,30,50,80,100 and $b_{k}\neq 0$ for k=1,30. We also assume smaller effects for the second components than the first, that is we assume that the first components are more informative than the second. In particular, we set $a_{1}=3.5$, $a_{30}=2$, $a_{50}=a_{80}=2.5$, $a_{100}=1.5$ and $b_{1}=1.5$, $b_{30}=1$. From the first plot at the bottom of Figure 9, we see that when both components are predictive, the entire pair is included into the model with a probability almost 1, even though the second components are attributed smaller effects than the first. For the pairs $\left(u_{50},v_{50}\right)$ and $\left(u_{100},v_{100}\right)$, the probability that the entire pair is selected is overestimated, yet smaller than the probability than only the first component is selected. The same is not true for pair $\left(u_{80},v_{80}\right)$ of which the probability of selecting the entire pair exceeds, even though marginally, the probability of selecting the single component. This overestimation of the inclusion probabilities for the noninformative components is counterbalanced by assigning them a zero effect, as can be seen from the marginal effects plot at the bottom of Figure 9. We see from this plot that, in general, the model manages to estimate the true magnitude of the various effects. A more clear picture of the relative importance/contribution of each predictor component is given in the last plot of Figure 9 that shows the estimates of combined summaries. The most prominent points in this plot correspond to the informative components of the five predictive pairs, which suggests that PS provides an adequate summary for assessment of the true impact of the component variables in the model. Results from extending this scenario to the situation where we additionally have correlations between the predictive pairs are presented in the Appendix.

## 5 DISCUSSION

In this work, we addressed the problem of isotope cluster selection through a Bayesian model formulation. Results from applying the Bayesian selection model to the pancreatic cancer data showed rather strong evidence in favor of specific isotope clusters being associated with the class outcome while most often a limited number of about 15 isotope clusters was selected by the model as a sufficient subset for separating the two groups. In addition to the isotope selection, the model formulation allows for assessment of the added‐value of the shape source over and above the intensity source through the employment/application of an additional layer of shape selection within the isotope cluster selection. To perform the shape selection we make the explicit assumption that this type of information is complementary to the intensity information which, in the model fitting process, can be formulated as shape being included in the model on the condition that its corresponding intensity is already selected or will be selected to the model alongside with shape. This assumption, and hence this “asymmetrical” manner in which we select/deselect shape, is based on our prior knowledge from previous work that the overall intensity level is a superior source of predictive information as compared to shape. The main outcome with regards to assessing the added‐value of shape was that most shape measures were associated with effects of much smaller magnitude as compared to intensity (conditional on the assumed model), regardless of their inclusion or not in the model, suggesting that intensity alone provides all the required information for separating the two groups.

We compared results from our Bayesian variable selection approach with results obtained with an alternative frequentist variable selection approach based on a stability selection strategy that utilizes sparse‐group lasso (SS‐SGL). We describe how stability selection can be employed in order to obtain “selection probabilities” that may serve as a measure of confidence for the variables selected by sparse‐group lasso. Intuitively, the selection probabilities can be considered equivalent to the inclusion probabilities generated by our Bayesian variable selection approach. It should be noted however that the Bayesian variable selection method is structurally—and in terms of objectives—different from SS‐SGL due to the underlying assumptions linked with prior knowledge on the relative predictive power of each individual source. Because of this structural difference, the selection probabilities resulting from SS‐SGL do not have the same interpretation as posterior probabilities and, moreover, the definition of the selection probabilities is different from that of the inclusion probabilities generated with the Bayesian approach due to the statistics being different. To overcome the interpretational differences, we restricted our comparison to the evaluation of results obtained using each method with respect to the achieved isotope selection and classification performance. Results from the real data analysis showed that the Bayesian approach selects lower dimensional models to describe the data and leads to a more consistent variable selection compared to SS‐SGL. Moreover, classification results suggested that the Bayesian approach can lead to greater accuracy (as indicated by the considerably smaller error‐rates) compared to SS‐SGL, justifying thus the choice of incorporating prior knowledge in the analysis by making problem‐specific assumptions.

Variable selection methods have seen many applications within the Bayesian statistics field. Among these, a popular method for performing variable selection is via spike‐and‐slab priors (George & McCulloch, 1993). Sparsity is achieved in this case by placing a mixture prior on the regression effect of each predictor consisting of a “spike” either exactly at or around zero, corresponding to exclusion of a specific variable from the model, and a flat “slab,” corresponding to inclusion of the variable to the model. Variable selection using spike‐and‐slab priors is performed by introducing latent binary indicator variables for each predictor to denote whether the predictor belongs to the slab or spike part of the prior. Priors are placed on the binary indicator variables to encourage sparsity. MCMC sampling is often used to fit this type of models in which fixed prior parameters are frequency specified in order to reduce the computational burden. A variant of the slab‐and‐spike priors was considered by Dellaportas et al. (2000, 2002) who proposed including the binary indicator variables $\gamma_{j}$ in the likelihood so that the variables that do not contribute to the linear predictor, which is now of the form $\beta_{0}+\sum_{j=1}^{k}\gamma_{j}\beta_{j}X_{j}$, drop off. In our Bayesian selection approach we used a reversible‐jump implementation in which the level of sparsity is controlled through a prior on the model (isotope) dimension. This allows us, on the one hand, to estimate the optimal dimensionality of the isotope clusters predictor set, and on the other, to assess the additional value of shape at the isotope cluster level, through the Metropolis–Hastings acceptance ratios, which account for inclusion or exclusion of shape to or from the model.

In a recent paper, Rodríguez‐Girondo et al. (in press) proposed a frequentist two‐step approach for assessing the augmented predictive value of a secondary source on top of a primary source based on a sequential double cross‐validation procedure. Apart from the selective nature of our Bayesian model, a key difference between this sequential approach and our Bayesian selection method is that the latter takes explicitly into account the pairing structure of our data and could be extended to deal also with more complex grouping data structures such as triplets, quadruples etc. Moreover, in contrast to the sequential approach, our Bayesian approach can be used not solely to evaluate whether there is additional predictive information in the secondary source (shape) after correcting for the primary (intensity) source but also to address the question of where this extra information, if any, comes from. This is essentially achieved through the “asymmetrical” layer of shape selection within the isotope cluster selection that evaluates—and estimates—for each unique isotope cluster the relative contribution of each shape measure to classification.

An alternative way of implementing the double‐layered selection of isotope clusters, on the one hand, and shape measures within the isotope cluster configuration, on the other, would be to restrict the parameter space by modifying the prior specification on the regression coefficient parameters. More specifically, rather than assuming Normal priors for the intensity and shape effects we may constrain the regression coefficients to be within the set of values {−1,0,1}. In this way, we replace calibration of the regression coefficients with selection by restricting the effect to be absent, present‐negative or present‐positive. Moreover, by assuming prespecified values for the magnitude of the regression effects, no further assumptions, for instance on the distribution of the variances for the intensity and shape effects, are needed. A limitation of this particular prior specification is that simple conjugate updating rules are no longer applicable. This suggests that calculation of the acceptance probabilities would require actual estimation of the integrated likelihood functions of the old and newly proposed models that could increase the computational time considerably.

We restricted our discussion to intensity‐shape combinations in which we considered simple linear effects for both intensity and shape summary measures, mainly due to the fact that such linear rules can be calibrated and interpreted with relative ease in practical application. In reality however the relationship between the class outcome and the two predictor sources—as well as the relationship between the intensity source and the shape source —may be more complex. In fact, it would be interesting to consider more complex effect structures, for example by including also quadratic terms for intensity and shape as well as interactions between intensity and shape into the model in addition to the linear effects. Allowing for this flexibility in the model could potentially improve the predictive capacity of intensity and shape integration and give us the chance to learn more about the interplay between these two different types of information. The idea of considering more complex structures in order to capture the true relationships in the data, in the hope that this will result in improved predictions and/or more thorough inference, is a promising topic for future research. Preliminary results from an exploratory analysis showed that including quadratic as well as interaction terms in a univariate model (i.e., a model with only one intensity predictor and one shape predictor fitted separately for each isotope cluster), could lead to improvement of the model fit, as compared to univariate models with only linear terms, for a considerable number of clusters.

An undesired feature of the proposed Bayesian selection approach is that it tends to overselect shape components even in the cases where there is no true effect. This tendency of the model is more pronounced for isotope clusters that are selected with high overall isotope probability of inclusion. The overestimation of the inclusion probabilities for the non‐informative shape components is partly counterbalanced by the calibration of the marginal regression coefficients for shape that were effectively zero for almost all shape measures that were included to the model with high frequency. Overestimation of inclusion probabilities is actually a known deficiency of reversible‐jump estimation procedures for (linear) models, yet not discussed/identified in the related literature.

For posterior inference, we proposed a posterior summary that combines the information that can be extracted from the inclusion probability estimates and the marginal regression effect estimates as a way to deal with the “inflated” shape selection. Results from both the real data analysis and a simulation study showed that the “combined” posterior summary gives a reasonable assessment of the relative contribution of shape in separating the two groups when combined with intensity. A more formal solution to this problem would be to modify the assumptions on the distribution of the variance for the shape effects (or the previously mentioned −1,0,1 based model proposal that decouples effect estimation from variable selection). For example, instead of an Inverse Gamma distribution for the shape variance we could assume a Uniform distribution in order to shift the values of the variance distribution to larger values. This would result in more conservative shape selection as, for higher values of $\sigma_{b}$, the acceptance probability for adding a shape measure becomes smaller while the acceptance probability for removing a shape measure becomes larger. The drawback however with this solution is that conjugate updating is not applicable for this family of distributions. Another alternative could be to use a more general and flexible prior specification for the variance of the intensity and shape effects so that the model can adapt to the data at hand. A solution toward this direction could be to assume independent—across isotopes—Inverse Gamma priors with different hyperparameters for the intensity and shape variances with additional priors placed on these hyperparameters. This prior specification could give us more flexibility and control in borrowing strength across the shape measures as compared to setting the hyperparameters of the Inverse Gamma priors to fixed values.

## CONFLICT OF INTEREST

The authors have declared no conflict of interest.

## Supporting information

Appendix

Appendix

Supplementary Material
