# Supplementary material for: Bayesian variable selection logistic regression with paired proteomic measurements
Source: Biom J. 2018 Jun 25;60(5):1003–20. doi: 10.1002/bimj.201700182 (PMC6175404; doi:10.1002/bimj.201700182)
Supplement: Supplementary file 1 — Appendix [file BIMJ-60-1003-s001.pdf]

Appendix: Supplementary Material

A. Calculation of acceptance probabilities

Let T be the total number of isotopes in our data set and $k = 0, 1, ..., k_{max}$, the model (isotope) dimension. Within each model dimension k, isotopes may be included as either singletons (intensity) or couples (intensity & shape) such that the isotope dimension k equals the number of intensity singletons k_I plus the number of intensity-shape couples k_C. We use the following 6 move types:

We assume that within each isotope dimension, all models are equally likely. We choose the proposal probabilities as

$$b^I_{(k=0)} = b^C_{(k=0)} = d^I_{(k=k_{max}=k_I)} = d^C_{(k=k_{max}=k_C)} = c^{I\to C}_{(k=k_{max}=k_I)} = c^{C\to I}_{(k=k_{max}=k_C)} = 1/2,$$

$$d^I_{(0<k_I,k_C<k_{max}=k)} = d^C_{(0<k_I,k_C<k_{max}=k)} = c^{C\to I}_{(0<k_I,k_C<k_{max}=k)} = c^{I\to C}_{(0<k_I,k_C<k_{max}=k)} = 1/4,$$

$$b^I_{(k_I=0<k_C<k_{max})} = b^C_{(k_I=0<k_C<k_{max})} = d^C_{(k_I=0<k_C<k_{max})} = c^{C\to I}_{(k_I=0<k_C<k_{max})} = 1/4,$$

$$b^I_{(k_C=0<k_I<k_{max})} = b^C_{(k_C=0<k_I<k_{max})} = d^I_{(k_C=0<k_I<k_{max})} = c^{I\to C}_{(k_C=0<k_I<k_{max})} = 1/4,$$

$$b^I_{(k=k_{max})} = b^C_{(k=k_{max})} = d^I_{(k_I=0)} = d^C_{(k_C=0)} = c^{C\to I}_{(k_C=0)} = c^{I\to C}_{(k_I=0)} = 0$$

and

$$b^I_k = b^C_k = d^I_k = d^C_k = c^{C\to I}_k = c^{I\to C}_k = 1/6$$

in all other cases.

The acceptance probability for a proposal move from a model with parameters $\boldsymbol{\theta}$ to a new model with parameters $\boldsymbol{\theta'}$ is given by

$$a = \min\left\{1, \frac{P(D|\boldsymbol{\theta'})}{P(D|\boldsymbol{\theta})}\frac{p(\boldsymbol{\theta'})}{p(\boldsymbol{\theta})}\frac{q(\boldsymbol{\theta}|\boldsymbol{\theta'})}{q(\boldsymbol{\theta'}|\boldsymbol{\theta})}\right\},$$

the ratio of 1) marginal likelihoods, 2) priors and 3) proposal distributions.

For the prior distribution of $\boldsymbol{\theta}$ we use a discrete uniform specification of the form

$$p(\boldsymbol{\theta}) = \binom{T}{k}^{-1}\binom{k}{k'}^{-1}\frac{1}{k_{max}+1}$$

where k' is either one of k_I or k_C. The first two terms in the equation ensure that each model with isotope dimension k and intensity singleton dimension k_I or intensity-shape couple dimension k_C is equally likely. In this way we assume that, given k, any set of potential isotope predictors is found by sampling k isotope clusters from the candidate set $\mathcal{T}$ without replacement. The last term assumes that each possible isotope dimension $k \in \{0, 1, ..., k_{max}\}$ is equally likely.

Before we show how to calculate the prior and proposal ratios for all 6 move types based on the description in [1], we explain the notion of the proposal ratio. This involves understanding both the BIRTH and the reverse DEATH moves. Suppose we add a new intensity singleton component, then we use the proposal density $q(\boldsymbol{\theta'}|\boldsymbol{\theta}) = b_{I(k)}/(T-k)$. This consists of the

probability of attempting this particular birth move and the probability of choosing this particular new component which can be done in $T - k$ ways. The probability of proposing the reverse move is $q(\boldsymbol{\theta'}|\boldsymbol{\theta}) = d_{I(k+1)}/(k_I + 1)$ which is the provability of proposing the death of an intensity singleton and then of choosing the proposed component as the the one to remove. The ratio of marginal likelihoods BF can be calculated according to the equations given in [1].

BIRTH MOVE

Birth of an I singleton

$$k \to k+1, \quad k_I \to k_I + 1, \quad k_C \to k_C$$

$$\text{prior ratio} = \frac{p(\boldsymbol{\theta'})}{p(\boldsymbol{\theta})} = \frac{\dfrac{1}{\binom{T}{k+1}}}{\dfrac{1}{\binom{T}{k}}} \frac{\dfrac{1}{\binom{k+1}{k_I+1}}}{\dfrac{1}{\binom{k}{k_I}}} \frac{\dfrac{1}{k_{max}+1}}{\dfrac{1}{k_{max}+1}} = \frac{k_I + 1}{T - k}$$

$$\text{proposal ratio} = \frac{q(\boldsymbol{\theta}|\boldsymbol{\theta'})}{q(\boldsymbol{\theta'}|\boldsymbol{\theta})} = \frac{\dfrac{d_{I(k+1)}}{k_I + 1}}{\dfrac{b_{I(k+1)}}{T - k}} = \frac{T - k}{k_I + 1} \frac{d_{I(k+1)}}{b_{I(k)}}$$

$$R = \frac{p(\boldsymbol{\theta'})}{p(\boldsymbol{\theta})} \frac{q(\boldsymbol{\theta}|\boldsymbol{\theta'})}{q(\boldsymbol{\theta'}|\boldsymbol{\theta})} = \frac{d_{I(k+1)}}{b_{I(k)}}$$

$$\text{If } k = 0 \ \to \ \frac{d_{I(k+1)}}{b_{I(k)}} = \frac{1}{6}, \ \text{else } \frac{d_{I(k+1)}}{b_{I(k)}} = 1$$

Birth of an IS pair

$$k \to k+1, \quad k_I \to k_I, \quad k_C \to k_C + 1$$

$$\text{prior ratio} = \frac{p(\boldsymbol{\theta'})}{p(\boldsymbol{\theta})} = \frac{\dfrac{1}{\binom{T}{k+1}}}{\dfrac{1}{\binom{T}{k}}} \frac{\dfrac{1}{\binom{k+1}{k_C+1}}}{\dfrac{1}{\binom{k}{k_C}}} \frac{\dfrac{1}{k_{max}+1}}{\dfrac{1}{k_{max}+1}} = \frac{k_C + 1}{T - k}$$

$$\text{proposal ratio} = \frac{q(\boldsymbol{\theta}|\boldsymbol{\theta'})}{q(\boldsymbol{\theta'}|\boldsymbol{\theta})} = \frac{\dfrac{d_{C(k+1)}}{k_C + 1}}{\dfrac{b_{C(k+1)}}{T - k}} = \frac{T - k}{k_C + 1} \frac{d_{C(k+1)}}{b_{C(k)}}$$

$$R = \frac{p(\boldsymbol{\theta'})}{p(\boldsymbol{\theta})} \frac{q(\boldsymbol{\theta}|\boldsymbol{\theta'})}{q(\boldsymbol{\theta'}|\boldsymbol{\theta})} = \frac{d_{C(k+1)}}{b_{C(k)}}$$

$$\text{If } k = 0 \ \rightarrow \ \frac{d_{C(k+1)}}{b_{C(k)}} = \frac{1}{6}, \text{ else } \frac{d_{C(k+1)}}{b_{C(k)}} = 1$$

DEATH MOVE

Death of an I singleton

$$k \rightarrow k-1, \quad k_I \rightarrow k_I - 1, \quad k_C \rightarrow k_C$$

$$\text{prior ratio} = \frac{p(\boldsymbol{\theta'})}{p(\boldsymbol{\theta})} = \frac{\frac{1}{\binom{T}{k-1}}}{\frac{1}{\binom{T}{k}}} \frac{\frac{1}{\binom{k-1}{k_I-1}}}{\frac{1}{\binom{k}{k_I}}} \frac{\frac{1}{k_{max}+1}}{\frac{1}{k_{max}+1}} = \frac{T-k+1}{k_I}$$

$$\text{proposal ratio} = \frac{q(\boldsymbol{\theta}|\boldsymbol{\theta'})}{q(\boldsymbol{\theta'}|\boldsymbol{\theta})} = \frac{\frac{b_{I(k-1)}}{T-k+1}}{\frac{d_{I(k)}}{k_I}} = \frac{k_I}{T-k+1} \frac{b_{I(k-1)}}{d_{I(k)}}$$

$$R = \frac{p(\boldsymbol{\theta'})}{p(\boldsymbol{\theta})} \frac{q(\boldsymbol{\theta}|\boldsymbol{\theta'})}{q(\boldsymbol{\theta'}|\boldsymbol{\theta})} = \frac{b_{I(k-1)}}{d_{I(k)}}$$

$$\text{If } k = k_{max} \ \rightarrow \ \frac{b_{I(k-1)}}{d_{I(k)}} = \frac{1}{6}, \text{ else } \frac{b_{I(k-1)}}{d_{I(k)}} = 1$$

Death of an IS pair

$$k \rightarrow k-1, \quad k_I \rightarrow k_I, \quad k_C \rightarrow k_C - 1$$

$$\text{prior ratio} = \frac{p(\boldsymbol{\theta'})}{p(\boldsymbol{\theta})} = \frac{\frac{1}{\binom{T}{k-1}}}{\frac{1}{\binom{T}{k}}} \frac{\frac{1}{\binom{k-1}{k_C-1}}}{\frac{1}{\binom{k}{k_C}}} \frac{\frac{1}{k_{max}+1}}{\frac{1}{k_{max}+1}} = \frac{T-k+1}{k_C}$$

$$\text{proposal ratio} = \frac{q(\boldsymbol{\theta}|\boldsymbol{\theta'})}{q(\boldsymbol{\theta'}|\boldsymbol{\theta})} = \frac{\frac{b_{C(k-1)}}{T-k+1}}{\frac{d_{C(k)}}{k_C}} = \frac{k_C}{T-k+1} \frac{b_{C(k-1)}}{d_{C(k)}}$$

$$R = \frac{p(\boldsymbol{\theta'})}{p(\boldsymbol{\theta})} \frac{q(\boldsymbol{\theta}|\boldsymbol{\theta'})}{q(\boldsymbol{\theta'}|\boldsymbol{\theta})} = \frac{b_{C(k-1)}}{d_{C(k)}}$$

$$\text{If } k = k_{max} \ \rightarrow \ \frac{b_{C(k-1)}}{d_{C(k)}} = \frac{1}{6}, \text{ else } \frac{b_{C(k-1)}}{d_{C(k)}} = 1$$

CHANGE MOVE

Change of an I singleton to an IS pair

$$k \rightarrow k, \quad k_I \rightarrow k_I - 1, \quad k_C \rightarrow k_C + 1$$

$$\text{prior ratio} = \frac{p(\boldsymbol{\theta'})}{p(\boldsymbol{\theta})} = \frac{\dfrac{1}{\binom{T}{k}}}{\dfrac{1}{\binom{T}{k}}} \frac{\dfrac{1}{k_{max}+1}}{\dfrac{1}{k_{max}+1}} = 1$$

$$\text{proposal ratio} = \frac{q(\boldsymbol{\theta}|\boldsymbol{\theta'})}{q(\boldsymbol{\theta'}|\boldsymbol{\theta})} = \frac{\dfrac{c_{RS(k)}}{k_C+1}}{\dfrac{c_{AS(k)}}{k_I}} = \frac{k_I}{k_C+1} \frac{c_{RS(k)}}{c_{AS(k)}}$$

$$R = \frac{p(\boldsymbol{\theta'})}{p(\boldsymbol{\theta})} \frac{q(\boldsymbol{\theta}|\boldsymbol{\theta'})}{q(\boldsymbol{\theta'}|\boldsymbol{\theta})} = \frac{k_I}{k_C+1} \frac{c_{RS(k)}}{c_{AS(k)}}$$

$$\text{If } k = k_I \ \rightarrow \ \frac{c_{RS(k)}}{c_{AS(k)}} = \frac{1}{6}, \text{ else } \frac{c_{RS(k)}}{c_{AS(k)}} = 1$$

Change of an IS pair to an I singleton

$$k \rightarrow k, \quad k_I \rightarrow k_I + 1, \quad k_C \rightarrow k_C - 1$$

$$\text{prior ratio} = \frac{p(\boldsymbol{\theta'})}{p(\boldsymbol{\theta})} = \frac{\dfrac{1}{\binom{T}{k}}}{\dfrac{1}{\binom{T}{k}}} \frac{\dfrac{1}{k_{max}+1}}{\dfrac{1}{k_{max}+1}} = 1$$

$$\text{proposal ratio} = \frac{q(\boldsymbol{\theta}|\boldsymbol{\theta'})}{q(\boldsymbol{\theta'}|\boldsymbol{\theta})} = \frac{\dfrac{c_{AS(k)}}{k_I+1}}{\dfrac{c_{RS(k)}}{k_C}} = \frac{k_C}{k_I+1} \frac{c_{AS(k)}}{c_{RS(k)}}$$

$$R = \frac{p(\boldsymbol{\theta'})}{p(\boldsymbol{\theta})} \frac{q(\boldsymbol{\theta}|\boldsymbol{\theta'})}{q(\boldsymbol{\theta'}|\boldsymbol{\theta})} = \frac{k_C}{k_I + 1} \, \frac{c_{AS(k)}}{c_{RS(k)}}$$

$$\text{If } k = k_C \;\rightarrow\; \frac{c_{AS(k)}}{c_{RS(k)}} = \frac{1}{6}, \text{ else } \frac{c_{AS(k)}}{c_{RS(k)}} = 1$$

B. Additional simulation scenarios

We explore the behaviour of the Bayesian variable selection approach under the additional situations in which we have low, moderate or high correlations between the pairs and/or between the predictive components within and across pairs. As in the simulation examples presented in the paper, we generate data to have the same number of patients as in the pancreatic cancer data set but smaller dimensionality. We generate 200 variables in total such that half of them represent the first and the other half the second components of 100 paired measurements. In contrast to the scenaria presented in the paper, here we do not assume independence between pairs/components.

We simulate the binary outcome data according to the logistic model

$$\begin{aligned} \text{logit}\,(p) &= \beta_0 + \boldsymbol{\beta z} \\ &= \beta_0 + \boldsymbol{au} + \boldsymbol{bv} \end{aligned} \tag{1}$$

where $\boldsymbol{a}$ and $\boldsymbol{b}$ are 100-dimensional vectors containing the first and second component effects on the class outcome. To induce associations between the predictor components and the outcome, we draw binary response variables from a Bernoulli distribution with

$$p = \frac{1}{1 + e^{-(\beta_0 + \boldsymbol{au} + \boldsymbol{bv})}} \tag{2}$$

For both components u and v of each pair we simulate data from the normal distribution $N(0, 2.5^2)$. In order to induce the desired correlations between the chosen variables, we multiply the selected variables with the upper triangular matrix obtained from the Cholesky decomposition of the desired correlation matrix R.

We consider 6 additional scenarios which are extensions of the last two scenarios presented in the main paper. Apart from the number of true, non-zero effects, and their magnitude, here, we also vary the number of correlated components and the magnitude of their correlations. In all 6 scenarios we use $k_{max} = 50$, $\alpha = \beta = 1$ for the hyperparameters of the Gamma distribution and $\tau_a = \tau_b = 1$ while we vary the values of the non-zero elements of β from 1 to 4.5.

In the first 5 additional scenarios (4rh-8th), we assign non-zero effects to both components of the first pair and to the first component of the last pair. In scenarios 4 until 6, we introduce high, moderate and high correlations between the first components of the two predictive pairs. In particular, the correlation between the two components is set to 0.75, 9.50 and 0.25 for the 4th, 5th and 6th scenarios respectively. We set the effect for the two components of the first pair equal to 2.5 and the effect of the first component of the last pair equal to 1.5. Figure 1 show estimates of the inclusion probabilities (left plots), marginal effects (middle plots) and combined summaries (right plots) across 200,000 simulations for the 4th (first row), 5th (middle row) and 6th (last row) scenarios. In general, we observe similar results with those from the second scenario where we assume independence between all components, that is, the method detects all predictive components and estimates the true effects with high accuracy although it slightly overestimates the inclusion probability of the non-informative component of the last pair. In the case of high correlation between the first components of the two predictive pairs, the method identifies the first component as the only predictive variable and assigns all the effect to that particular component.

In the 7th scenario we consider the case in which the components within the pair are moderately correlated. We should note that this is a situation which we do not expect to occur in reality since estimation of shape is invariant under transformations of the overall intensity level and hence shape and intensity measures are conceptually independent from each other. The effects for the two components of the first pair, as before, are equal to 2.5 and the effect of the first component of the last pair equal is 1.5. We set the correlation between the first and second components equal to 0.6. Results from this scenario are shown in the first row of Figure 2, from which we observe that despite this correlation between the two components the method consistently selects both predictive components to be included in the model, although we see that some of the effect of the u_1 is goes into the that of v_1.

In the 8th scenario, we introduce correlations between all 3 predictive components (first and second components of the first pair and first component of the last pair). More specifically, we set corr(u_1,v_1), corr(u_1,u_{100}) and corr(u_{100},v_1) equal to 0.65, 0.50 and 0.25 respectively. We set the effects of u_1 and v_1 to 4.5 and the effect of u_{100} to 3.5. Results shown in the middle row of Figure 2 suggest that the method correctly identifies the predictive components and accurately estimate the effects. Inflation of the inclusion probability for the non-predictive component of the last pair is observed also in this scenario.

Finally, we increase the number of pairs with both components discriminative to two and the number of pairs with only the first component discriminative to three such that $a_k \neq 0$ for $k = 1, 30, 50, 80, 100$ and $b_k \neq 0$ for $k = 1, 30$. We set $a_1 = 4.5$, $a_{30} = 3.5$, $a_{50} = a_{80} = 2.5$, $a_{100} = 2$ and $b_1 = 2.5$, $b_{30} = 1.5$. We introduce correlations between the u_1, u_30, u_50 and u_100 such that

$$R = \begin{matrix} 1 & 0.65 & 0.50 & 0.25 \\ 0.65 & 1 & 0.40 & 0.20 \\ 0.50 & 0.40 & 1 & 0.10 \\ 0.25 & 0.20 & 0.10 & 1 \end{matrix}$$

Results based on this scenario are shown in the last row of Figure 2. We see that when both components are predictive, the entire pair is included into the model with a probability almost 1, even though the second components are attributed smaller effects than the first. Again here we observe overestimation of the inclusion probabilities for the non-informative components of the predictive pairs which is counterbalanced by assigning them a zero effect. The most prominent points in the last plot of Figure 2 correspond to the informative components of the 5 predictive pairs, which suggests that PS provides an adequate summary for assessment of the true impact of the component variables in the model.

References

[1] Denison, D G T and Holmes, C C and Mallich, B K and Smith A F M. Bayesian Methods for Nonlinear Classification and Regression. *Wiley series in probability and statistics* 2002.

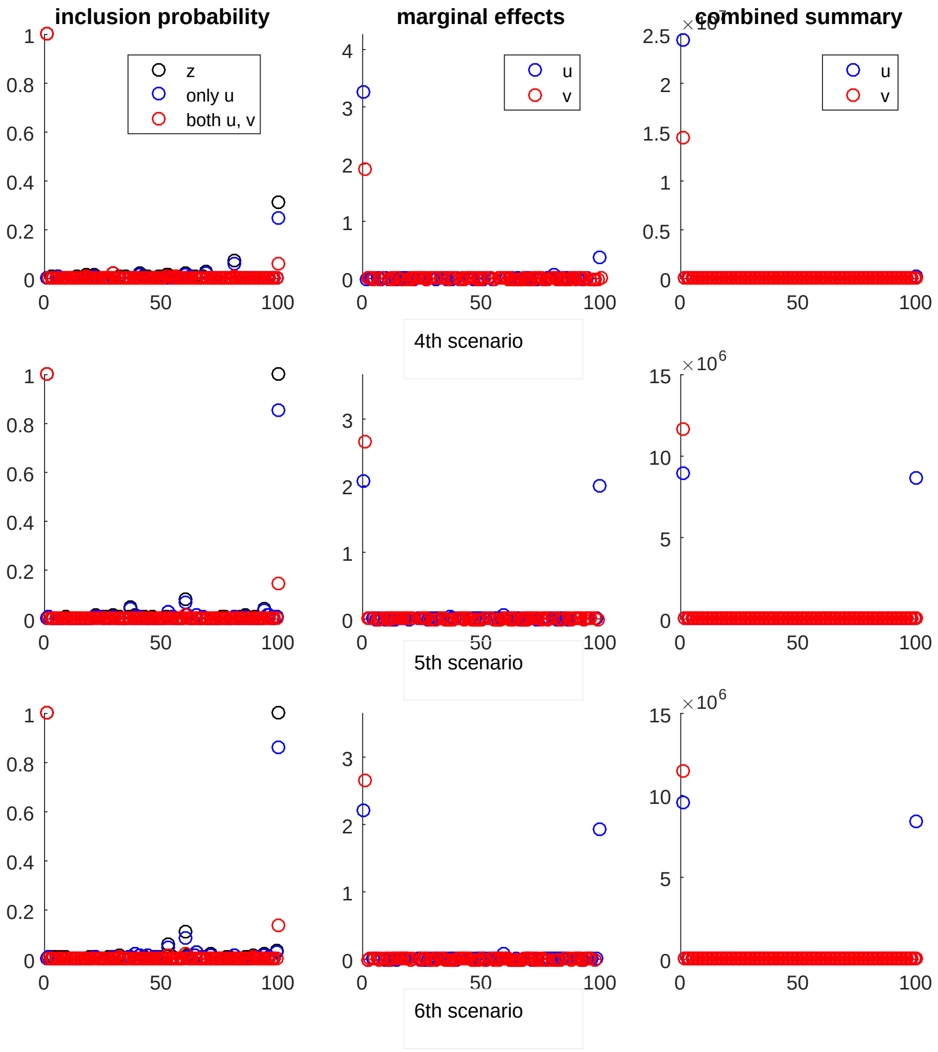

Figure 1: Inclusion probabilities (left plots), marginal regression effects (middle plots) and combined posterior summaries (right plots) of the first (blue points) and second (red points) components of the pairs for the 4th (top plots), 5th (middle plots) and 6th (bottom plots) scenarios.

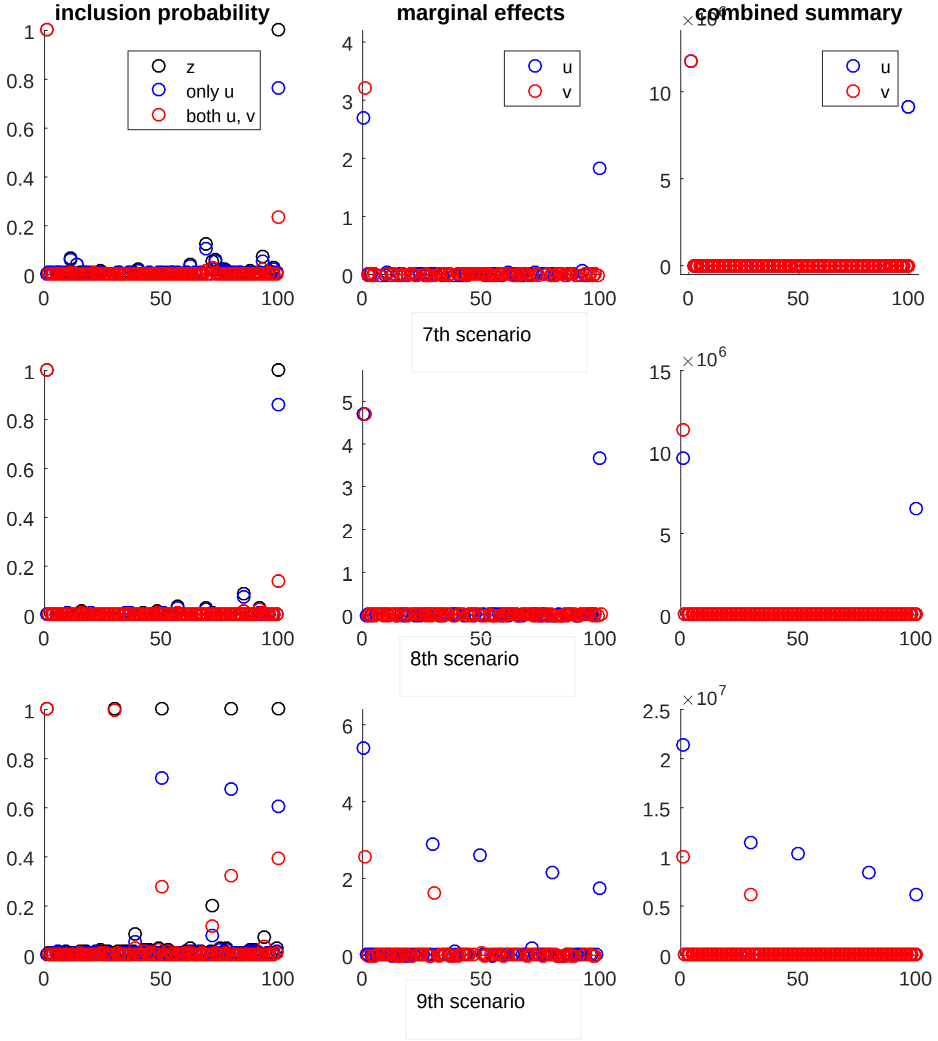

Figure 2: Inclusion probabilities (left plots), marginal regression effects (middle plots) and combined posterior summaries (right plots) of the first (blue points) and second (red points) components of the pairs for the 7th (top plots), 8th (middle plots) and 9th (bottom plots) scenarios.
